# The Folding process of Human Profilin-1, a novel protein associated with familial amyotrophic lateral sclerosis

**DOI:** 10.1038/srep12332

**Published:** 2015-07-31

**Authors:** Edoardo Del Poggetto, Fabrizio Chiti, Francesco Bemporad

**Affiliations:** 1 Department of Experimental and Clinical Biomedical Sciences, Section of Biochemistry, University of Florence, Viale Morgagni 50, I-50134, Florence, Italy

## Abstract

Human profilin-1 is a novel protein associated with a recently discovered form of familial amyotrophic lateral sclerosis. This urges the characterization of possible conformational states, different from the fully folded state, potentially able to initiate self-assembly. Under native conditions, profilin-1 is monomeric and possesses a well-defined secondary and tertiary structure. When incubated at low pH or with high urea concentrations, profilin-1 remains monomeric but populates unfolded states exhibiting larger hydrodynamic radius and disordered structure, as assessed by dynamic light scattering, far-UV circular dichroism and intrinsic fluorescence. Refolding from the urea-unfolded state was studied at equilibrium and in real-time using a stopped-flow apparatus. The results obtained with intrinsic fluorescence and circular dichroism indicate a single phase without significant changes of the corresponding signals before the major refolding transition. However, such a transition is preceded by a burst phase with an observed increase of ANS fluorescence, which indicates the conversion into a transiently populated collapsed state possessing solvent-exposed hydrophobic clusters. Kinetic analysis reveals that such state has a conformational stability comparable to that of the fully unfolded state. To our knowledge, profilin-1 is the first example of an amyloid-related protein where folding occurs in the absence of thermodynamically stable partially folded states.

Human profilin-1, also named platelet profilin, is one of the four known human profilins which acts as a complex regulator of the assembly of monomeric actin (G-actin) into filaments (F-actin)^1^,^2^. Multiple properties allow profilin-1 to play such a central role in F-actin assembly: (i) profilin-1 catalyses the exchange of ADP with ATP increasing the pool of profilin-1 bound to G-actin-ATP, which is the most represented pool for F-actin growth; (ii) profilin-1 inhibits filament nucleation; (iii) it allows growing of F-actin filaments only at their barbed ends, as opposed to the pointed ends; (iv) it competes effectively with thymosin-β4 for G-actin binding, thus favouring F-actin elongation as G-actin-thymosin-β4 complexes are incompetent for filament growth; (v) profilin-1 also binds poly-Pro sequences in various cytoskeletal proteins, thus affecting the formation of the cytoskeletal net; (vi) profilin-1 can inhibit F-actin polymerisation when present at high concentrations^1^,^2^.

From a structural perspective, profilin-1 is a 139-residue protein whose three-dimensional structure has been solved with both NMR^3^ (PDB entry 1PFL) and X-ray crystallography (unpublished results; PDB entry 1FIK). Both structures show that it consists of an antiparallel, 7-stranded β-sheet packed between three amphipathic α-helices, two on one side of the sheet and one on the other side (Fig. 1A). This fold, which is named *profilin-like* according to the Structural Classification of Proteins (SCOP), is shared by roughly 25 structural families of proteins, such as the GAF-domain, the flavin-binding PAS domain, the sedlin and the YNR034W-A-like families.

Very recently, profilin-1 has been associated with a familial form of amyotrophic lateral sclerosis (fALS): three amino acid substitutions of profilin-1, namely C71G, M114T and G118V, have been found to be causative pathogenic mutations for fALS, whereas a fourth mutation, namely E117G, has been proposed to be a moderate risk factor for developing ALS^4^. It was shown that overexpression of mutant profilin-1 in neuroblastoma N2A cells or primary motor neurons results in the formation of cytosolic, ubiquitin-positive profilin-1 aggregates^4^. The formation of intracellular inclusions by the same protein bearing the pathogenic mutations suggests that protein aggregation is a key process of the disease, highlighting the importance of elucidating its fundamental aspects to gain insight into the pathogenesis of the disorder. In this regard, fALS forms associated with mutant profilin-1 are similar to other identified forms of fALS, where aggregation also occurs for the protein bearing the mutations; examples include fALS associated with mutations of superoxide dismutase 1^5^, RNA binding protein FUS^6^, vesicle associated membrane protein associated protein B^7^, C9orf72^8^,^9^ and TAR DNA-binding protein 43^10^. Even sporadic forms of the disease, or fALS cases where the mutant protein does not aggregate, are associated with formation of intracellular inclusions of a protein, namely the TAR DNA-binding protein 43^11^,^12^.

Protein aggregation is known to proceed through the formation of a misfolded intermediate, which can be either native-like and possess a well-defined fold on the native side of the major free energy barrier for unfolding or a partially folded state on the unfolded side of the same barrier^13^. It is therefore important to study the folding process of profilin-1 from the fully unfolded to the fully folded state. In fact, protein folding studies of amyloidogenic proteins have often revealed partially folded states, as shown for β2-microglobulin^14^,^15^,^16^, lysozyme^17^, transthyretin^18^ and cystatin C^19^. Many of these partially folded states have been shown to be prone to aggregate, therefore offering important mechanistic insight into their processes of protein aggregation^14^,^16^,^17^,^19^. The lack of knowledge of the folding process of profilin-1 limits our current understanding of the aggregating properties of this protein.

In this work we have purified human profilin-1 from an *Escherichia coli* expression system, checked that the resulting protein is pure, non-aggregated and adopting a native structure and studied its folding process from the urea-unfolded state to the fully folded conformation. When (un)folding was studied at equilibrium and by means of kinetic experiments the protein was found to fold through an apparent two-state mechanism, with no significant tryptophan packing and secondary structure formation before the major transition for folding. We will also show, however, that the refolding of the protein is preceded by a hydrophobic collapse involving the transient formation of a species lacking persistent structure. Such a state represents a well defined partially folded state that is unstable and thermodynamically indistinguishable from the unfolded state.

## Results

### Purified human profilin-1 adopts a native structure

**Figure 1:**
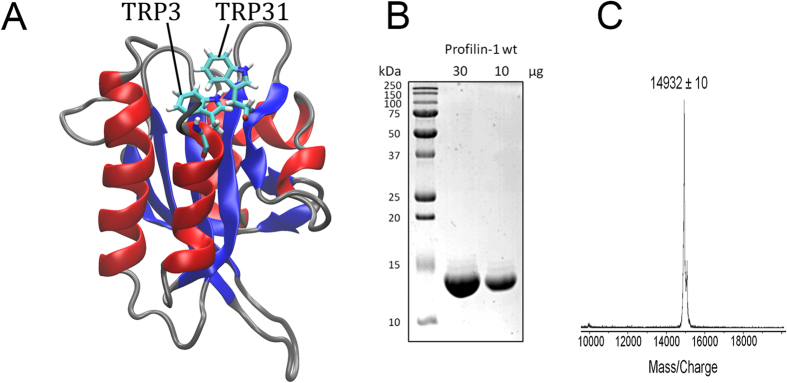
Profilin-1 structure and purification. (**A**) Three-dimensional structure of native profilin-1. The structure was determined by multidimensional heteronuclear NMR spectroscopy^3^ and drawn with VMD for win-32 from PDB entry 1PFL. Red, blue and grey colors indicate α-helices, β-strands and loops, respectively. The positions of Trp residues are shown as sticks (Trp3, Trp31). (**B**) SDS-PAGE of profilin-1 after purification. An aliquot of the protein after purification (30 μg, left) and following a 3-fold dilution (10 μg, right) were loaded. Protein purity, calculated with ImageJ software, was 90–95%. (**C**) MALDI mass spectrometry analysis of profilin-1 after purification. The expected molecular weight for human profilin-1 devoid of the initial methionine residue is 14923 Da (Uniprot code P07737, residues 2–140).

Human profilin-1 (Uniprot code P07737, residues 2-140) was purified as described in the *Methods* section. The sample containing the expected purified protein was analysed using SDS-PAGE and MALDI mass spectrometry. The two SDS-PAGE electrophoretic profiles obtained using two different amounts of protein sample (30 and 10 μg of loaded protein) showed a single band at ca. 14 kDa, with only weak bands, if any, of other proteins at different molecular weights (Fig. 1B). When the sample was subjected to MALDI mass spectrometry, a single peak at 14932 ± 10 Da was detected (Fig. 1C), in agreement with the expected molecular weight of the protein devoid of the initial methionine (14923 Da).

**Figure 2:**
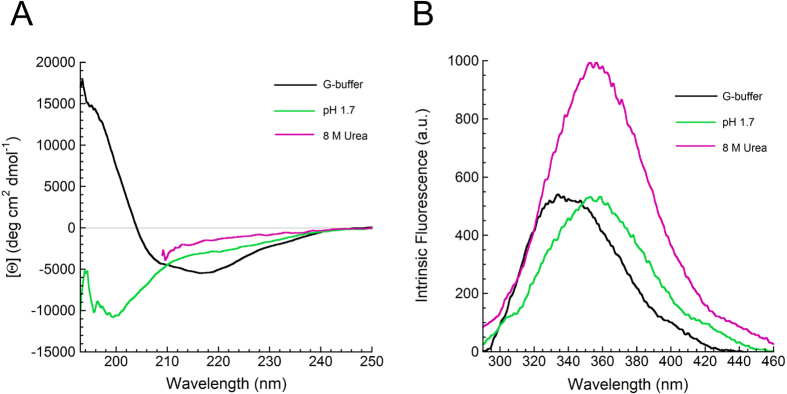
Characterization of Profilin-1 structure under different conditions. (**A**) Far-UV CD spectra and (**B**) intrinsic fluorescence emission spectra of profilin-1 samples under three conditions: G-buffer (black), pH 1.7 (green) and in 8 M urea (magenta).

Despite the use of 8 M urea in the purification procedure, dialyzed and renatured profilin-1 was previously found to exhibit normal functional properties^20^. In order to assess whether the purified protein adopts a native structure, far-UV circular dichroism (CD) spectra of the protein in G-buffer at pH 7.3, in a solution at pH 1.7 and in another solution containing 8 M urea were acquired (Fig. 2A). The far-UV CD spectrum recorded in G-buffer is indicative of a mixed α + β protein and is identical to that previously recorded for native profilin-1^21^. By contrast, the spectrum recorded at pH 1.7 featured a minimum at 200 nm and a broad shoulder at 212–225 nm, indicating a substantially unfolded protein with some residual α-helical or β-sheet structure. The far-UV CD spectrum acquired in 8 M urea, recorded from 250 to 208 nm due to the large and prohibitive absorbance of urea below this range of wavelengths, showed mean residue ellipticity values close to zero, indicating an even more unfolded ensemble (Fig. 2A).

We also acquired intrinsic fluorescence spectra of the purified protein under the same experimental conditions (Fig. 2B). The spectrum acquired in G-buffer showed two overlapping peaks at 325–335 nm and 340–350 nm, respectively, probably arising from different chemical environments and different degrees of solvent exposure for the two tryptophan residues of the protein (Trp3 and Trp31). These wavelengths are, however, typical of a compact folded structure as flexible and solvent exposed tryptophan residues in unfolded proteins typically exhibit a maximum fluorescence at 350–357 nm. The protein at pH 1.7 shows a single peak at 355 nm. As the emission peak of N-acetyl-l-tryptophanamide -a molecule that mimics tryptophan side chain- was not shown to undergo any change as pH varies from 7.0 to 2.0^22^, we ascribed this spectrum to a largely unfolded structure and to a similar environment for the two tryptophan residues. The spectrum in 8 M urea also has a single peak at ca. 356 nm, again indicating a largely unfolded structure. The latter spectrum shows higher fluorescence; this is probably due to quenching of the emitted fluorescence by residues that are located near the tryptophan moieties in the native structure but become distant following denaturation. The larger, albeit small, red-shift of the tryptophan fluorescence peak and the higher overall intrinsic fluorescence indicates that the urea-unfolded ensemble of profilin-1 is more disordered than the acid-unfolded state.

### Human profilin-1 adopts a soluble, monomeric state

**Figure 3:**
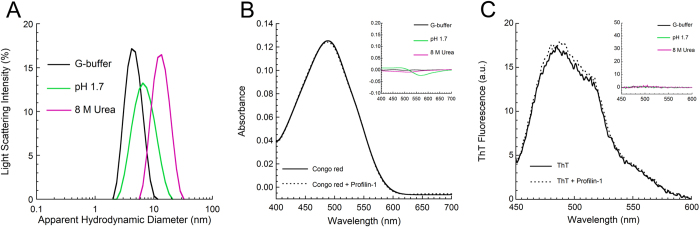
Assessment of Profilin-1 self-assembly. (**A**) Size distributions of profilin-1 samples obtained with DLS under three conditions: G-buffer (black), pH 1.7 (green) and in 8 M urea (magenta). (**B**) CR optical absorption spectra measured in the absence (continuous line) and presence (dashed line) of profilin-1 in G-buffer. The inset shows the difference between the CR absorption spectrum recorded in the presence and that recorded in the absence of the protein under the same conditions listed in panel (**A**). Color code as in panel (**A**). (**C**) Fluorescence emission spectra of ThT in the presence (dashed line) and absence (continuous line) of profilin-1 in G-buffer. The inset shows the difference between the ThT emission spectrum recorded in the presence and that recorded in the absence of the protein under the same conditions listed in panel (**A**). Color code as in panel (**A**).

Following the observation that human profilin-1 has a significant propensity to aggregate^4^, we checked whether the purified protein is soluble or aggregated. The size distribution of the protein sample in G-buffer, obtained using dynamic light scattering (DLS), showed the presence of a single species with an apparent hydrodynamic diameter of 4.7 ± 0.2 nm (Fig. 3A). This value is consistent with a hydrodynamic diameter of 4.3 nm determined from the NMR or X-ray structures and is significantly lower (p <0.001) than that expected for a dimer of the native protein (5.7 nm) or a native tetramer (7.16 nm). By contrast, the size distributions of the protein unfolded at low pH and in urea showed apparent hydrodynamic diameters of 7.3 ± 0.7 nm and 13.8 ± 1.4 nm, respectively (Fig. 3A). As the expected hydrodynamic diameter for the unfolded state of a 139 residue long protein is 7.0 nm^23^, these two latter values indicate a largely unstructured ensemble under either condition.

Protein aggregates possessing β-sheet structure are known to bind to amyloid diagnostic dyes such as Thioflavin-T (ThT) and Congo red (CR), which change their spectral properties as a consequence of these interactions. All three profilin-1 samples in G-buffer, at pH 1.7 and in 8 M urea were found to leave the spectral properties of ThT and CR unaffected. As an example, Fig. 3B,C show the representative spectra of CR and ThT, respectively, acquired in the presence and absence of native profilin-1 in G-buffer. For all three profilin-1 samples in G-buffer, at pH 1.7 and in 8 M urea the difference spectra are substantially flat (insets in Fig. 3B,C). Overall, these data indicate that our protein sample in G-buffer contains a purified profilin-1 protein that appears native and monomeric and that treatment of the protein either at pH 1.7 or in 8 M urea unfolds the protein in the absence of any detectable aggregation.

### Profilin-1 is characterised by a highly cooperative unfolding at equilibrium

**Figure 4:**
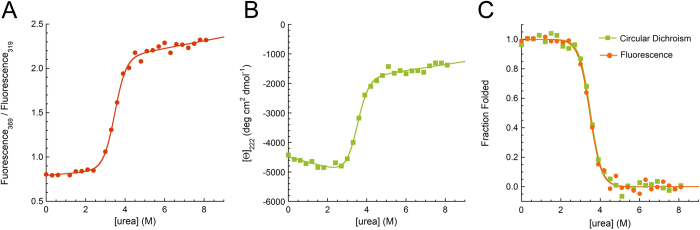
Urea-induced equilibrium denaturation curves of profilin-1. (**A**) Ratio between fluorescence emitted at 369 and 319 nm. (**B**) Mean residue ellipticity per residue at 222 nm. (**C**) Fraction of folded protein calculated from the fluorescence data reported in panel (**A**) (orange circles) and from the CD data shown in panel (**B**) (green squares). The fraction folded was calculated with equation (1). In all panels, the solid line represents the best fits of the data points to the equation edited by Santoro and Bolen^25^.

In order to investigate the conformational stability of profilin-1 we set out to perform urea-induced denaturation curves in G-buffer, at 25 °C. To this aim, we exploited two different and complimentary spectroscopic probes. Tryptophan fluorescence provides information about tertiary structure and reports on the compaction of the hydrophobic core. Furthermore, tryptophan 3 was shown to be indirectly involved in the interaction with phosphatidylinositol 4,5-bisphosphate^21^ and both residues are located in the poly-L-pro binding site^24^. Far-UV CD is a probe of secondary structure content. The plots of tryptophan fluorescence and CD *versus* urea concentration were obtained at various wavelengths and all of them showed a single cooperative transition located between 2.5 and 4.5 M urea, suggesting that profilin-1 equilibrium denaturation occurs through a two-state process in the absence of intermediate states. Figure 4A,B show two representative plots obtained at well-defined wavelengths. All curves were analysed with the method provided by Santoro and Bolen^25^. Analysis of the fluorescence curves (Fig. 4A) yielded values of 26.3 ± 1.9 KJ mol^−1^ and 7.4 ± 0.6 KJ mol^−1^ M^−1^ for the free energy change upon denaturation in the absence of denaturant 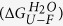 and for the dependence of free energy change on urea concentration (*m*_eq_), respectively. The ratio between these two values gave a concentration of middle denaturation (*C*_m_) of 3.57 ± 0.04 M. The same analysis, carried out with the CD data, yielded a 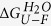 of 26.3 ± 2.2 KJ mol^−1^, an *m*_eq_ value of 7.5 ± 0.7 KJ mol^−1^ M^−1^ and a *C*_m_ of 3.50 ± 0.03 M. The fraction folded was calculated as reported in the Methods section (equation (1)) and plotted *versus* urea concentration in Fig. 4C for two representative fluorescence and CD plots. This analysis revealed that the plots obtained with fluorescence and CD are overlapped and that, consequently, they describe the same transition. Overall, the weighted average of the values obtained with the two analyses are 26.3 ± 2.2 KJ mol^−1^, 7.5 ± 0.7 KJ mol^−1^ M^−1^ and 3.54 ± 0.04 M for 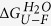, *m*_eq_ value and *C*_m_, respectively. The thermodynamic values measured here in G-buffer at pH 7.3 are in reasonable agreement with those previously reported under slightly different conditions. The *C*_m_ was shown to be 3.5 M at pH 8.1^21^; using CD and fluorescence, average values of 25.4 ± 4.6 KJ mol^−1^, 7.8 ± 1.4 KJ mol^−1^ M^−1^ and 3.35 M were gauged at pH 6.5–7.0 for 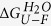, *m*_eq_ value and *C*_m_, respectively^24^.

### Folding and unfolding monitored in real-time reveal single-exponential kinetics

**Figure 5:**
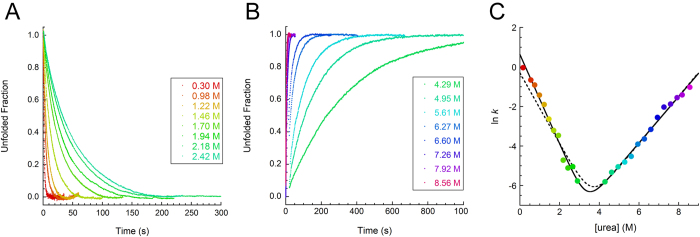
Investigation of Profilin-1 folding. (**A**,**B**) Plot showing the unfolded fraction *versus* time during profilin-1 refolding (**A**) or unfolding (**B**) experiments carried out at different urea concentrations. Color scale is shown in the legend. (**C**) Natural logarithm of profilin-1 folding / unfolding rate constant as a function of urea concentration. The solid line through the data represents the best fit of the data points to the equation proposed by Jackson and Fersht^26^. Dotted line represents the best fit of the data to the equation for a two-state transition by forcing the curve through the folding rate in the absence of denaturant calculated using the unfolding rate constant extrapolated at 0 M urea and the conformational stability measured from equilibrium denaturation experiments.

In order to pinpoint the folding mechanism of profilin-1, folding and unfolding kinetics of the protein were investigated using a stopped-flow device coupled to fluorescence detection. Refolding of profilin-1 was investigated by diluting the protein, initially unfolded in 5 M urea, down to final urea concentrations ranging from 0.3 to 2.9 M urea. The process involved a decrease in fluorescence, which was complete in ca. 5 s in 0.3 M urea (the smallest urea concentration we could reach). After the end of this refolding phase, no further changes in fluorescence could be detected, ruling out the possibility of proline isomerism. Kinetic traces obtained at different urea concentrations were analysed with equation (2) and normalized to the unfolded fraction. Representative traces are shown in Fig. 5A. Profilin-1 unfolding was studied by diluting the folded protein to final urea concentrations ranging from 4.29 to 8.56 M urea. Results showed that tryptophan fluorescence increased as the protein underwent denaturation. As for refolding, data were analysed with equation (2) and normalized to the unfolded fraction. Representative traces are shown in Fig. 5B.

We obtained the rate constants *k* for folding (*k*_F_) and unfolding (*k*_U_) from best fits of experimental traces to equation (2) and we built the plot of the natural logarithm of the apparent *k versus* urea concentration (Fig. 3C). This plot, conventionally referred to as chevron plot, revealed a linear dependence of ln(*k*_F_) and ln(*k*_U_) on urea concentration and was consequently analysed with equation (3)^26^. The folding rate constant in the absence of denaturant (*k*_F_^H2O^) was 1.87 ± 0.30 s^−1^. The extrapolated unfolding rate constant in the absence of denaturant (*k*_U_^H2O^) was 1.86 (±0.40) · 10^−5^ s^−1^. The dependence of −*RT* ln(*k*_F_) and *RT* ln(*k*_U_) on urea concentration (*m*_*k*F_ and *m*_*k*U_) were 5.6 KJ mol^−1^ M^−1^ and 2.9 KJ mol^−1^ M^−1^, respectively. These values allowed the calculation of equilibrium parameters from kinetic data. The obtained values were 28.5 ± 2.0 KJ mol^−1^, 8.5 ± 0.7 KJ mol^−1^ M^−1^ and 3.36 ± 0.40 M for 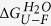 *m* value and *C*_*m*_, respectively. Such results were consistent, within experimental error, with those obtained from equilibrium unfolding experiments (see above). Furthermore, we used the conformational stability obtained by equilibrium experiments and the extrapolated unfolding rate in the absence of denaturant to calculate the expected folding rate for a two-state folder (dashed line in Fig. 5C). As transient formation of intermediate states is typically expected to slow down refolding, one should expect a calculated folding rate higher than the experimental value. In the case of profilin-1, the obtained theoretical value was 0.74 s^−1^, which is smaller than the experimental one.

Refolding kinetics experiments were also performed under the same buffer conditions, but with profilin-1 concentrations ranging from 0.03 to 0.16 mg/mL. The lack of dependence of ln(*k*_F_) on protein concentration (not shown) shows that profilin-1 does not undergo any transient aggregation effect^27^,^28^. Consequently, kinetic analysis of profilin-1 folding/unfolding is compatible either with a two-state model or with the transient formation of a partially folded ensemble having the same free energy as the fully unfolded state.

The β-Tanford value (β_T_) of profilin-1, calculated as the ratio *m*_*k*F_/*m*_eq_, is 0.65. This value, which theoretically ranges from 0 to 1, describes the relative position of the folding transition state along the reaction coordinate and is proportional to the compaction of the transition state^29^,^30^. A survey in the kineticDB database showed that β_T_ values range from 0.52 to 0.95 with a median value of 0.72 in the case of two-state folders and from 0.44 to 0.94 with a median of 0.70 in the case of multi-state folders^31^. Thus, the transition state of profilin-1 can be described as an ordinary transition state, slightly disordered compared to the average.

### The major folding transition is preceded by hydrophobic chain collapse

**Figure 6:**
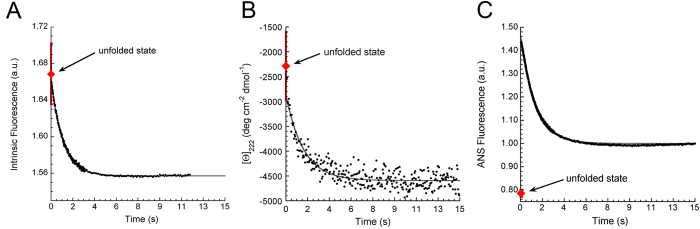
Refolding traces of profilin-1 obtained in the presence of 0.45 M urea. The folding process was followed with (**A**) intrinsic fluorescence, (**B**) far-UV CD at 222 nm and (**C**) ANS fluorescence. In all panels the red diamond represents the signal of the fully unfolded protein extrapolated from values measured at high urea concentrations. The solid lines represent the best fits of experimental data to equation (2).

We further investigated the conformational ensemble populated at the beginning of profilin-1 refolding by measuring a set of structure-related spectroscopic signals at the beginning of the kinetic traces (time zero) and comparing the obtained values with those corresponding to the unfolded and folded states. For each spectroscopic probe, the signal of the folded state was measured at the end of the refolding process. The signal of the unfolded ensemble under native conditions was obtained from linear extrapolation of the equilibrium signal measured at the end of unfolding kinetics experiments performed at different high urea concentrations (not shown). Figure 6A shows the plot obtained in the case of tryptophan fluorescence. Similarly to Fig. 5A, refolding of profilin-1 in 0.45 M urea involves a decrease in fluorescence. The *k*_F_ value under such conditions was 0.95 ± 0.30 s^−1^. The extrapolated emission at the beginning of refolding is consistent, within experimental error, with fluorescence of the unfolded state at the same urea concentration.

The same conclusions could be drawn when the experiment was repeated using [Θ] at 222 nm as a probe of secondary structure (Fig. 6B). Following dilution of unfolded profilin-1 into a refolding buffer to a final denaturant concentration of 0.5 M, [Θ] at 222 nm decreased from a value of −2860 ± 200 to −4590 ± 200 deg cm^2^ dmol^−1^, with a *k*_F_ value of 0.66 ± 0.30 s^−1^. While the latter value corresponds to the [Θ] value of the native state at 222 nm (Fig. 2A), the former is consistent with the [Θ] value measured for the unfolded protein under the same conditions (−2290 ± 680 deg cm^2^ dmol^−1^).

A different behaviour was observed when 8-Anilinonaphthalene-1-sulfonic acid (ANS) binding was used as a spectroscopic probe (Fig. 6C). Neither folded nor unfolded profilin-1 were found to bind ANS, as fluorescence emitted by the dye in the presence of the protein was similar to the signal of the blank solution at all urea concentrations (not shown). However, when ANS fluorescence was followed in real-time during refolding of profilin-1 in the presence of 0.45 M urea, the ANS fluorescence at the beginning of the process was significantly higher than those of the unfolded and folded states and then decreased with a *k*_F_ value of 0.80 ± 0.30 s^−1^. This suggested the presence of a hidden burst phase, occurring within the dead-time of our stopped-flow experiments, wherein profilin-1 induced ANS fluorescence jumps to a value which is ca. 46% higher than the signal of the folded state. This behaviour suggested that urea-unfolded profilin-1 collapses into an ensemble of conformations possessing hydrophobic clusters exposed to the solvent and that this ensemble is spectroscopically different from the fully folded and unfolded states, albeit thermodynamically similar to the unfolded state.

## Discussion

In the present manuscript we purified human profilin-1 and we first characterized the structural properties of the native, acid- and urea-unfolded states. Our results show that the native protein exhibits a far-UV CD spectrum consistent with a α  +  β fold, has tryptophan fluorescence at wavelengths typical of fully or partially buried side chains, again typical of a folded state, a hydrodynamic diameter consistent with that of a folded protein of this size. The results also show that the protein sample is not aggregated, as reported by the absence of species with a large size in the DLS size distribution and by the spectroscopic signals of amyloid reporting dyes. By contrast, dilution of the protein into solutions containing high denaturant or acid concentrations induces the disruption of secondary and tertiary structure, as indicated by far-UV CD and fluorescence spectroscopies, although the protein remains soluble and does not aggregate, as assessed by DLS measurements and by the spectroscopic signals of amyloid reporting dyes. When comparing these two ensembles, we could detect that the urea unfolded state exhibits a higher extent of disorder. Indeed, the CD spectrum of the urea unfolded state has a lower signal in the 210–225 nm region of wavelengths, where negative peaks or shoulders arising from α-helical and/or β-sheet structure are generally evident, if present. Moreover, the increased intrinsic fluorescence of the urea unfolded state and the larger red-shift of its fluorescence peak suggest that solvent exposure of the tryptophan indole moieties is only partial in the acid-unfolded state. Consistently, DLS measurements show a larger hydrodynamic radius for urea unfolded profilin-1, suggesting an open conformation with larger solvent accessible surface.

Our investigation of the equilibrium and kinetics of folding/unfolding of profilin-1 at pH 7.3 and 25 °C is compatible with either a two-state model or a multi-state model involving the transient formation of a highly unstable conformation. The equilibrium urea unfolding curve consists of a single highly cooperative transition and the curves monitored with far-UV CD and intrinsic fluorescence superimpose when normalized to the fraction folded. Both observations suggest that profilin-1 unfolding is a single-step process where only the fully folded and fully unfolded states are significantly populated at equilibrium. The linear dependence of ln(*k*_F_) on denaturant concentration, the consistence between thermodynamic parameters calculated from equilibrium and kinetic data and the agreement between the initial spectroscopic signals recorded at “time zero” in the kinetic traces probed with far-UV CD and intrinsic fluorescence and those of the unfolded state extrapolated from the high denaturant concentrations corroborate the idea that any partially folded state transiently formed during the dead-time of the stopped-flow refolding kinetics would have the same energy as the fully unfolded state. This conclusion holds true even at small denaturant concentrations, where partially folded states are generally more stable.

In contrast with the results described above, our ANS binding studies suggested that a chain collapse, leading to hydrophobic clusters exposed to the solvent, occurs during the dead-time of our refolding experiments, immediately after removal of urea and before the observed exponential phase of folding. As neither folded nor unfolded states of profilin-1 are able to bind the dye, these data suggest the transient formation of a collapsed state. Remarkably, the *k*_F_ obtained from best fits of the trace shown in Fig. 6C to equation (2) (0.80 ± 0.30 s^−1^) is in agreement with the folding rate under the same conditions (0.675 s^−1^), obtained from the chevron plot shown in Fig. 5C. This allows the possibility of a change in the folding pathway induced by ANS to be ruled out. Notwithstanding that, the chevron plot indicates that this conformational state is energetically indistinguishable from the fully unfolded state. In addition, the kinetic traces probed with far-UV CD and intrinsic fluorescence indicate that the collapse of unfolded profilin-1 is not accompanied by the formation of secondary structure and native-like burial of tryptophan side chains. Consequently, we believe that the observed ANS burst phase corresponds to a conformational rearrangement of unfolded profilin-1 following denaturant removal and that this process consists of the collapse of protein segments structurally distant from the two tryptophan side chains and not involving formation of secondary structure.

Other protein systems have been shown to fold through an initial chain collapse which precedes folding but does not involve structure formation. For example, folding of the SH3 domain of bovine phosphatidylinositol 3-kinase was shown initially to follow a two-state model. However, when the process was monitored in the sub-millisecond time scale, a chain collapse was found to occur within the first 150 μs of folding before the major structural transition^32^. Indeed, a jump in ANS fluorescence corresponding to an increase of about 20–30% of the ANS fluorescence of the unfolded protein takes place during the dead-time of stopped-flow folding kinetics. Förster resonance energy transfer (FRET) experiments revealed that the conformational ensemble populated at the end of such burst phase exhibited a non-specific (position independent) decrease of all intra-molecular distances^32^. The cold-shock protein from *Bacillus caldolyticus* (*Bc*-Csp) was proposed to fold very rapidly following a two-state model in the absence of intermediates^33^. A FRET study was carried out in which tryptophan residues were engineered as donors and single 5-(((acetylamino)ethyl)amino)naphthalene-1-sulfonate (AEDANS) moieties were added to the protein as acceptors. Results showed that about half of the shortening of the intra-molecular distances upon folding occurred before the rate-limiting step^33^. Similar conclusions were drawn for the adenylate kinase from *E. coli*, which undergoes fast collapse into an ensemble of compact structures where the local environment of surface probes seems to be native-like but secondary structure elements remain unfolded^34^. Many multi-state folding proteins exhibit a hydrophobic collapse preceding the major folding transition, including the W38FW44F double mutant of the protein barstar^35^, dihydrofolate reductase from *E. coli*^36^, bovine pancreatic RNase A^37^ and single-chain monellin from the tropical berry *Dioscoreophyllum cumminsii*^38^.

The identification of partially folded conformational states and, more generally, the characterization of folding pathways represents a crucial step towards the achievement of a complete understanding of the behavior of proteins in solution and the identification of possible aggregation-competent conformations. Folding and misfolding have been recognized as two sides of the same coin^39^ and amyloidogenic states have been identified either on the “folded” or on the “unfolded” side of the major kinetic barrier for folding^13^,^40^. Here, we characterized the folding pathway of profilin-1, a protein whose misfolding and deposition has been recently linked to fALS^4^ and we found that a collapsed state transiently forms before the major folding transition occurs. Such a state is thermodynamically unstable, meaning that it displays a conformational stability similar to that of the fully unfolded state. Based on the present dataset, it is not possible to assess whether the formation of such conformational ensemble represents a non-specific rearrangement of the polypeptide chain or rather a specific formation of a very unstable conformational ensemble. Importantly, however, we are showing here for the first time that a protein linked to a protein deposition disease folds without the accumulation of significantly stable intermediate states.

It is possible that the mutants associated with fALS show different features, which could explain why the wild-type protein has not been observed so far associated with protein deposits in ALS or other protein deposition diseases. The experimental information we collected here will serve as a starting point for further studies in these directions, which will describe the mechanism by which profilin-1 misfolding leads to the formation of proteinaceous deposits.

## Methods

### Protein expression

*Escherichia coli* BL21 (DE3) cells transformed with the pET-11d vector expressing the cDNA of the wt PFN-1 gene (kindly provided by C. Ampe) were grown overnight at 37 °C in LB medium with 100 μg/mL ampicillin under vigorous shaking. Cells were then diluted 10-fold into fresh medium and grown at 37 °C until optical density at 600 nm reached a value of 0.5–0.6. Expression was induced by the addition of 1 mM isopropyl-β-D-thiogalactoside (IPTG) from Inalco (Milano, Italy). Cells were then incubated for an additional 4 h and harvested by centrifugation at 2,800 × g for 15 min. Cells were resuspended with 50 ml of PBS buffer (20 mM sodium phosphate, 250 mM NaCl, 1 mM EDTA, 1 mM β-mercaptoethanol, 0.1 mM PMSF, pH 7.3) and stored at −80 °C.

### Protein purification

Cells were thawed at 37°, incubated for 30 min with 1 mg mL^−1^ lysozime in ice and then sonicated at 40 kHz (five cycles of 30 s each). After 45 min of centrifugation at 31,000 × *g,* the presence of profilin-1 in the supernatant was checked by SDS-PAGE using 18% acrylamide. Protein purification was carried out with a modified version of a previously reported protocol^41^, optimized to increase final yield. Briefly, cyanogen bromide-activated-Sepharose 4B resin (Sigma Aldrich, Saint Louis, MO, US, cat. n. C9142) was prepared with poly(L-Proline) having a molecular weight of 1000–10000 Da (Sigma Aldrich, Saint Louis, MO, US, cat. n. P2254) according to the protocol supplied by the manufacturer. The resulting poly(L-proline)-Sepharose resin was used to set up an affinity chromatographic column. The supernatant obtained after centrifugation of the cell lysate was applied on this column pre-equilibrated with PBS buffer at 4 °C. The column was then washed at 4 °C with 100 mL of G-buffer (20 mM Tris/HCl, 2 mM DTT, 0.1 mM CaCl_2_, 0.2 mM NaN_3_, pH 7.3). Profilin-1 was eluted from the column with G-buffer containing 8 M urea. In order to increase purification yield, the column was incubated overnight in G-buffer +8 M urea under gentle shaking. Two 40 mL eluate fractions were subjected to two cycles of dialysis against 3 liters of G-buffer for 48 h and concentrated using an ultrafiltration device and a 3000 Da cut-off membrane (Millipore, Billerica, MA) at 4 °C. The obtained solution contained pure protein in G-buffer and was stored at −80 °C. The purity of the protein was 90–95% as assessed by SDS-PAGE and Coomassie Blue staining. The identity of the purified protein as *wild-type* profilin-1 devoid of the initial methionine residue (Uniprot code P07737, residues 2–140) was checked using MALDI mass spectrometry.

### Far-UV CD spectroscopy

Far-UV CD spectra of samples of containing 0.3 mg mL^−1^ profilin-1 were acquired at 25 °C under three different conditions: (i) G-buffer, (ii) 20 mM HCl, pH 1.7 and (iii) G-buffer containing 8 M urea. Spectra were collected from 190 to 250 nm using a J-810 Spectropolarimeter from Jasco (Tokyo, Japan) equipped with a thermostated cell holder attached to a Thermo Haake C25P water bath (Karlsruhe, Germany). A 1 mm path-length cell (Hellma) was used. All spectra were blank subtracted and converted to mean residue ellipticity per residue [Θ].

In the case of urea-induced equilibrium denaturation, 28 samples containing 0.3 mg mL^−1^ profilin-1 and urea ranging from 0 to 8.1 M were prepared and analysed as described above. A plot of [Θ] at a given wavelength *versus* urea concentration was obtained for various wavelengths ranging from 215 to 225 nm. Each plot was analysed with a procedure of best fitting using the model by Santoro and Bolen^25^, which yielded the free energy difference between the unfolded and the native states in the absence of denaturant 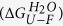, the dependence of Δ*G*_*U*−*F*_ on urea concentration (*m* value), and the midpoint of denaturation (*C*_m_). All values obtained at the 11 different wavelengths were utilized to provide the means and standard deviations. Plots were also converted to fraction folded (*F*_*n*_) *versus* urea concentration using the following equation:


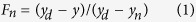


where *y* is the signal observed at a given urea concentration and *y*_*d*_ and *y*_*n*_ are the signals of denatured and native profilin-1, respectively, at the corresponding urea concentrations.

### Intrinsic Fluorescence

Intrinsic Fluorescence spectra of profilin-1 were recorded at a protein concentration of 0.03 mg mL^−1^ and 25 °C under three different conditions: (i) G-buffer, (ii) 20 mM HCl pH 1.7 and (iii) G-buffer containing 8 M urea. Spectra were measured between 290 and 460 nm using a PerkinElmer LS 55 spectrofluorimeter (Waltham, MA, USA) equipped with a thermostated cell holder attached to a Haake F8 water bath (Karlsruhe, Germany). Excitation wavelength was 280 nm. A 2 × 10 mm quartz cuvette was used.

In another set of experiments, 28 samples containing 0.03 mg mL^−1^ profilin-1 and urea at concentrations ranging from 0 to 8.1 M were prepared and analysed as described above. A plot of the ratio of the fluorescence values at two given wavelengths *versus* urea concentration was obtained for various wavelengths ranging from 360/328 to 370/318. The 11 plots were analysed as described above in the CD section.

### Dynamic light scattering

Size distribution analysis was performed at 25 °C with a Malvern Zetasizer Nano S DLS device (Malvern, Worcestershire, UK) using samples of 0.8 mg mL^−1^ profilin-1 under three conditions: (i) G-buffer, (ii) 20 mM HCl pH 1.7 and (iii) G-buffer containing 8 M urea. Each sample was centrifuged at 15700 × *g* for 10 minutes, filtered with 0.02 μm cut-off filters and analyzed considering the refraction index and viscosity of its dispersant. A 10 mm reduced volume plastic cell was used. Acquired data were compared with the theoretical hydrodynamic radius expected for folded and denatured polypeptide chains of *N* residues (*R*_*h*_^*folded*^ and *R*_*h*_^*denatured*^, respectively) calculated as 
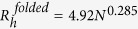
; 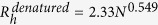, as previously reported^23^.

### CR absorbance

Spectra of CR (Sigma Aldrich, Saint Louis, MO, US, cat. n. C-6767) in the presence of profilin-1 were recorded at 25 °C under three different experimental conditions: (i) G-buffer, (ii) 20 mM HCl pH 1.7 and (iii) G-buffer containing 8 M urea. For each of the three conditions, spectra were acquired by adding 60 μL of 0.8 mg mL^−1^ profilin-1 to 440 μL of solution containing 20 μM CR (spectrum 1), by adding 60 μL of solution devoid of profilin-1 to 440 μL of solution containing 20 μM CR (spectrum 2), by adding 60 μL of 0.8 mg mL^−1^ profilin-1 to 440 μL of solution in the absence of CR (spectrum 3). Spectra were recorded from 400 to 700 nm using a Jasco V-630 spectrophotometer (Tokyo, Japan) and a 5 mm reduced volume quartz cell (Hellma). For each of the three conditions, the difference spectrum was obtained by subtracting spectrum 2 and spectrum 3 from spectrum 1.

### ThT Fluorescence

Samples containing 0.8 mg mL^−1^ profilin-1 were incubated at 25 °C under three experimental conditions: (i) G-buffer, (ii) 20 mM HCl pH 1.7 and (iii) G-buffer containing 8 M urea. An aliquot of 62 μL of each sample was mixed with 438 μL solution containing 25 μM ThT. In order to maintain the same concentrations of buffer components and denaturants in the samples, three different ThT-containing solutions were prepared, each reproducing one of the three conditions above described. Fluorescence spectra from 450 nm to 600 nm were then measured at 25 °C using a PerkinElmer LS 55 spectrofluorimeter (Waltham, MA, USA) equipped with a thermostated cell holder attached to a Haake F8 water bath (Karlsruhe, Germany), using an excitation wavelength of 440 nm. A 2 × 10 mm quartz cuvette was used. The ThT fluorescence spectrum obtained under the same conditions without profilin-1 was subtracted from that acquired in the presence of the protein.

### Folding and unfolding kinetics monitored with intrinsic fluorescence

Folding and unfolding kinetics were followed using a SFM-3 stopped-flow device coupled to a fluorescence detection system (Bio-Logic, Claix, France). An FC-08 quartz cuvette (from Bio-logic), an excitation wavelength of 280 nm and a band-pass filter were used to monitor emitted fluorescence above 320 nm. When the observed fluorescence did not reach equilibrium within 200 s, unfolding and refolding was studied with the Perkin Elmer LS 55 fluorimeter. An excitation wavelength of 280 nm and an emission wavelength of 369 nm were used, with a 2 × 10 mm quartz cuvette. All measurements were performed in G-buffer, pH 7.3, 25 °C, at final protein concentration of 0.04 mg mL^−1^. In another set of experiments, refolding was studied as profilin-1 concentration ranged from 0.03 to 0.16 mg mL^−1^, in G-buffer containing 0.5 M urea at pH 7.3 and 25 °C. The unfolding experiments were initiated by a 10-fold dilution of the native protein in 2 M urea into solutions containing urea at final concentrations ranging from 4.29 to 8.56 M. The refolding reactions were similarly initiated by a 10- to 20-fold dilution of the protein unfolded in 6 M urea into solutions containing final concentrations of urea between 0.30 and 2.89 M. The dead time ranged from 10.44 ms to 41.76 ms in the stopped-flow device, and was 15 s in the LS 55 fluorimeter. The unfolding and refolding traces were fitted to


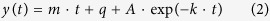


where *y*(*t*) is the fluorescence signal recorded as a function of time, *A* and *k* are the amplitude and rate constant, respectively, *q* is the equilibrium fluorescence at 0 s, and *m* is the slope of the equilibrium straight line. The obtained *k* values were plotted *versus* urea concentration and the obtained plot was analyzed with a modified version of the method proposed by Jackson and Fersht^26^:


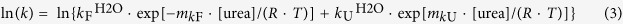


where *k*_F_^H2O^ and *k*_U_^H2O^ are folding and unfolding rate constants in the absence of denaturant, *m*_*k*F_ and *m*_*k*U_ are the dependences of −*RT* ln(*k*_F_^H2O^) and *RT* ln(*k*_U_^H2O^) on urea concentration, *R* is the ideal gas constant and *T* is the temperature in K.

### Folding kinetics monitored with ANS fluorescence

Folding of profilin-1 was monitored in the presence of ANS using the Bio-Logic stopped flow coupled to a fluorescence detection system. An FC-08 quartz cuvette (from Bio-logic), an excitation wavelength of 370 nm and a band-pass filter were used to monitor emitted fluorescence above 475 nm. The folding reaction was induced with a 10-fold dilution of 0.4 mg mL^−1^ profilin-1 denatured in 4.5 M urea into refolding buffer containing 110 μM ANS in G-buffer. Final conditions were 0.04 mg mL^−1^ profilin-1, 0.45 M urea, 99 μM ANS, G-buffer, pH 7.3, 25 °C. The dead time was 10.4 ms. The resulting ANS fluorescence trace was fitted to a single exponential function (equation (2)).

### Folding kinetics monitored with far-UV CD

Folding of profilin-1 was monitored with far-UV CD at 222 nm with a 1 nm bandwidth using an SFM-20 Bio-Logic stopped-flow device (Claix, France) coupled to a J-810 Spectropolarimeter from Jasco (Tokyo, Japan) and equipped with a 1 cm path-length cuvette (TC100-10T from Bio-logic). The folding reaction was initiated by a 10-fold dilution of 0.4 mg mL^−1^ profilin-1 denatured in 5 M urea into refolding buffer. Final conditions were 0.04 mg mL^−1^ profilin-1, 0.5 M urea, G-buffer, pH 7.3, 25 °C. The dead time of the experiment was 15 ms. 25 measurements were collected and the resulting traces were averaged to obtain a trace with a maximized signal-to-noise ratio. The signal of the buffered solution with no protein was subtracted from the averaged trace. The averaged trace was fitted to a single-exponential function (equation (2)).

## Additional Information

**How to cite this article**: Del Poggetto, E. *et al.* The Folding process of Human Profilin-1, a novel protein associated with familial amyotrophic lateral sclerosis. *Sci. Rep.* **5**, 12332; doi: 10.1038/srep12332 (2015).
